# The role of TGF-beta3 in cartilage development and osteoarthritis

**DOI:** 10.1038/s41413-022-00239-4

**Published:** 2023-01-02

**Authors:** Xinmei Du, Linyi Cai, Jing Xie, Xuedong Zhou

**Affiliations:** 1 State Key Laboratory of Oral Diseases, West China Hospital of Stomatology, Sichuan University, 610041 Chengdu, China; 2 National Clinical Research Center for Oral Diseases, West China Hospital of Stomatology, Sichuan University, 610041 Chengdu, China

**Keywords:** Bone, Multihormonal system disorders

## Abstract

Articular cartilage serves as a low-friction, load-bearing tissue without the support with blood vessels, lymphatics and nerves, making its repair a big challenge. Transforming growth factor-beta 3 (TGF-β3), a vital member of the highly conserved TGF-β superfamily, plays a versatile role in cartilage physiology and pathology. TGF-β3 influences the whole life cycle of chondrocytes and mediates a series of cellular responses, including cell survival, proliferation, migration, and differentiation. Since TGF-β3 is involved in maintaining the balance between chondrogenic differentiation and chondrocyte hypertrophy, its regulatory role is especially important to cartilage development. Increased TGF-β3 plays a dual role: in healthy tissues, it can facilitate chondrocyte viability, but in osteoarthritic chondrocytes, it can accelerate the progression of disease. Recently, TGF-β3 has been recognized as a potential therapeutic target for osteoarthritis (OA) owing to its protective effect, which it confers by enhancing the recruitment of autologous mesenchymal stem cells (MSCs) to damaged cartilage. However, the biological mechanism of TGF-β3 action in cartilage development and OA is not well understood. In this review, we systematically summarize recent progress in the research on TGF-β3 in cartilage physiology and pathology, providing up-to-date strategies for cartilage repair and preventive treatment.

## Introduction

The transforming growth factor-beta (TGF-β) superfamily includes TGF-β isoforms, activins/inhibins, nodal, anti-Müllerian hormone proteins, growth differentiation factors (GDFs), and bone morphogenetic proteins (BMPs), which play critical roles in embryogenesis and adult tissue homeostasis.^1,2^ The roles played by TGF-β isoforms, especially TGF-β1 and TGF-β2 in chondrocytes, have been extensively studied. However, few studies have focused on TGF-β3, which is equally important to cell proliferation, differentiation, migration and apoptosis.^3^ TGF-β3 is a 25-kDa homodimeric protein with a structure similar to its two homologous dimer-forming isoforms, TGF-β1 and TGF-β2.^4^ These three isoforms are potent agonists of chondrocyte differentiation and are generally applied in the field of tissue engineering and regenerative medicine. Moreover, they have specialized and overlapping effects on cartilage physiology and pathology.^5^

Articular cartilage, a smooth and wear-resistant tissue covering the surface of joints, supports and distributes applied loads. It is characterized by viscoelastic, anisotropic, tension–compression, and nonlinear properties, endowing cartilage with the ability to withstand extremely high mechanical stress.^6^ These characteristics rely on a set of cells composed of a small percentage of chondrocytes and an intricately organized matrix. The extracellular matrix (ECM) of hyaline cartilage constitutes as much as 98% of the cartilage volume and comprises primarily hyaluronan (HA), proteoglycans and collagens. Type II collagen accounts for 90% of all types of collagens in hyaline cartilage and confers tensile strength to the tissue.^7^ Proteoglycans form large aggregates by binding to spontaneously aggregating HA networks, endowing cartilage with compressive strength.^8^ Terminally differentiated chondrocytes constitute the only cell type in this tissue and account for ~5% of the total tissue volume. A small number of chondrocytes are sparsely dispersed within the matrix network and regulate cartilage health by controlling the dynamic balance between the anabolic and catabolic processes of ECM components.^9^ Without nerves, blood vessels, or a lymphatic system, cartilage fails to self-repair, and even minor lesions can result in progressive damage.^10^ The degradation of the cartilage structure ultimately leads to degenerative joint diseases such as osteoarthritis (OA), a painful condition that exerts a profound influence on individual physical health, including disability, the health care system, and society.^11^ Moreover, the repair of damaged cartilage with existing clinical techniques has been a challenge due to the extremely poor regenerative ability of this tissue.^12^ Therefore, it is essential to seek novel therapeutic targets for defective cartilage by elucidating the specific roles of various cytokines and growth factors in cartilage pathophysiology. In recent years, the profound roles of TGF-β3 in the anabolic processes of articular cartilage have been noticed by numerous researchers and may lead to new approaches to the restoration and reconstruction of cartilage defects.^13^

TGF-β3 contributes to cartilage physiology and pathology via the Smad-dependent pathway and a non-Smad pathway (herein, the Smad-independent pathway).^14^ The Smad family includes intracellular mediators that mediate TGF-β superfamily member signaling. Based on their structural and functional features, Smads are categorized into three major classes: receptor-regulated Smads (R-Smads), common mediator Smads (Co-Smads), and inhibitory Smads (I-Smads).^15^ As direct substrates of receptor kinases, R-Smads can be further classified into two subcategories, the Smad2/3 group and the Smad1/5/8 group, which each playing antagonistic roles during different stages of OA.^16^ The Smad-independent pathway is mediated by mitogen-activated protein kinases (MAPKs) (including extracellular regulated kinase (ERK), p38 kinase, c-Jun N-terminal kinase (JNK)), phosphoinositide 3-kinase (PI3K)/protein kinase B (AKT)/mammalian target of rapamycin (mTOR), and Rho-like GTPase.^17^ Overall, the Smad-dependent and Smad-independent pathways function independently but interact with each other to regulate cartilage development and maintenance. In summary, we review the recent progress in the understanding of TGF-β3 in cartilage development and the occurrence/progression of OA that is providing new targets for the treatment of cartilage diseases.

## Transforming growth factor-β3 overview

In the 1970s, polypeptides in the TGF-β family were first isolated and named sarcoma growth factor (SGF) by de Larco and Todaro.^18^ SGF is a mixture of two different compounds, TGF-β and transforming growth factor-alpha (TGF-α), that induces the malignant transformation of rat kidney fibroblasts.^19,20^ To date, the TGF-β superfamily primarily includes TGF-β isoforms, activins/inhibins, nodal, anti-Müllerian hormone proteins, GDFs and BMPs.

**Fig. 1:**
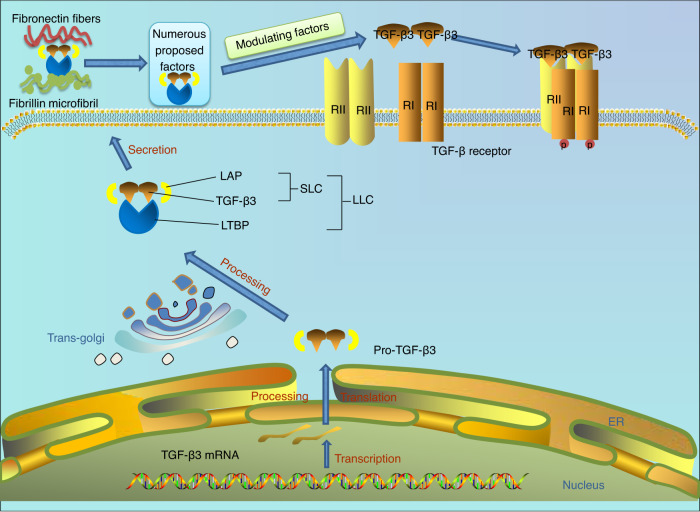
Schematic diagram showing the synthesis, proteolytic processing, secretion and activation of transforming growth factor-beta 3 (TGF-β3). TGF-β3 is produced through mRNA transcription and is transported to the cytoplasm. A pro-TGF-β3 homodimer consisting of the latency-associated peptide (LAP) and TGF-β3 is generated in the endoplasmic reticulum (ER) and then transported to the trans-Golgi network. In the Golgi apparatus, pro-TGF-β3 is further processed to form a small latent TGF-β3 complex (SLC). The SLC connects with the latent TGF-β3-binding protein (LTBP) and forms the resultant protein, the large latent TGF-β3 complex (LLC). LLC is secreted outside the cell and binds to various molecules in the extracellular matrix. Finally, the conformation of the LLC is change by modulating factors, leading to the release of the activated and mature TGF-β3. Activated TGF-β3 binds to corresponding receptors on the cell membrane

Three TGF-β isoforms are encoded by distinct genes located on different chromosomes. The active form is a homodimer consisting of two polypeptide chains connected by a disulfide bond.^21,22^ The biological activities of the three TGF-β isoforms are precisely modulated via four steps: synthesis, proteolytic processing, secretion, and activation (Fig. 1). Generally, TGF-β3 mRNA is produced in the nucleus and transported to the cytoplasm. The first polypeptide synthesized is pre-pro-TGF-β3, which carries N-terminal signal peptide (SP), a proregion called the latency-associated peptide (LAP), and a C-terminus. After SP removal and the dimerization of two polypeptides, a pro-TGF-β3 homodimer connected via three disulfide bonds is formed in the endoplasmic reticulum (ER) and is subsequently transported to the trans-Golgi network. In the Golgi apparatus, the LAP chains in pro-TGF-β3 are removed from the TGF-β3 molecule by a paired basic amino acid cleaving enzyme (PACE, also known as furin). Then, LAP and TGF-β3 are connected via noncovalent bonding, resulting in the formation of a small latent TGF-β3 complex (SLC). The SLC connects with the latent TGF-β3 binding protein (LTBP) to form the mature protein, the large latent TGF-β3 complex (LLC), which is transported from the cis pole of the Golgi apparatus secreted from a cell. After secretion, the trimeric structure of LLC prevents the interaction of TGF-β3 with its receptor, maintaining TGF-β3 in the inactive form.^23,24^ In addition, the TGF-β3 trimeric complex is attached to ECM components or the cell surface facilitated LTBP and glycoprotein-A repetitions predominant. The C-terminal end of LGBP interacts with fibrylin-1, and its N-terminal end covalently binds to fibronectin as catalyzed by a transglutaminase enzyme, providing a alternative mechanism to maintain TGF-β3 in the inactive form.^25^ Active TGF-β is released from LLC cells through the action of numerous ECM molecules, including proteases such as matrix metalloproteinase 2 (MMP2, also known as gelatinase A), matrix metalloproteinase 9 (MMP9, also known as gelatinase B), fibroblast growth factor 2 (FGF2), and reactive oxygen species.^26–28^ Hence, illuminating the activation mechanisms of latent TGF-β3 is of great importance. The RGD (Arg-Gly-Asp sequence) motif of TGF-β3 is recognized by αv integrins, which are necessary for integrin-mediated TGF-β3 activation.^29^ Once binding to integrins, LLC is conformationally changed via a nonproteolytic manner, leading to the inhibitory release and activation of mature TGF-β3.^30,31^ Among the members of the integrin family, integrin αvβ6 and αvβ8 are able of binding to LLC and activating TGF-β3.^32^

Since the processes of TGF-β3 synthesis, processing, secretion, and activation are elaborately regulated in chondrocytes, revealing the specific characteristics of TGF-β3 may lead to insights into cartilage repair and regeneration. Indeed, compared to other TGF-β isoforms, TGF-β3 shows subtle differences in the abovementioned process. For example, the precursor sequences of the TGF-β isoforms are largely dissimilar. The TGF-β3 precursor carries four potential N-glycosylation sites, while the precursors of TGF-β1 and TGF-β2 carry three of these sites, two of which are conserved in all three isoforms. In addition, the RGD peptide, which is recognized by αv integrins, is carried only by the TGF-β1 and TGF-β3 precursors. Therefore, αvβ6 integrin can cleave TGF-β3 ligand from LLC to activate TGF-β3.^33^ In addition, the three precursors differ in the number of cysteine residues; there are five in TGF-β3, six in TGF-β2, and three in TGF-β1 (which are conserved in all three isoforms).^34^ In addition, different TGF-β isoforms bind to different LTBPs. Evidence based on genetically mutated mice has shown that the association of TGF-β1 with LTBP is pivotal to extracellular TGF-β1 activity.^35^ Four isoforms of LTBP constitute a group of glycoproteins that interact with fibrillin microfibrils.^36^ LTBP-1 and LTBP-3 are thought to interact with all three TGF-β isoforms. LTBP-2 does not bind to any TGF-β isoform. LTBP-4 binds only to the LAP of TGF-β1.^37^ However, the specific role of each LTBP in the secretory mechanics of different TGF-β isoforms has not been clearly demonstrated.^38^

## TGF-β3 receptors

TGF-β receptors (TβRs) are mediators that mediate TGF-β signaling from the ECM to the intracellular space. TβRs are classified as a TGF-β type I receptor (TβRI), TGF-β type II receptor (TβRII), or TGF-β type III receptor (TβRIII) based on their sequence characteristics. TβRI proteins include activin receptor-like kinase 1–7 (ALK1-7). The TβRII protein subfamily consist of TβRII, the bone morphogenetic protein 2 (BMP2) receptor, activin type II receptor (ActRII), activin type I receptor (ActRI/Ib), and anti-Müllerian hormone receptor type 2 (AMHRII). TβRIII molecules include β-glucan and endoglin.^39–42^ TβRI and TβRII are structurally related transmembrane glycoproteins with three main sections: an extracellular ligand-binding site, a transmembrane sequence, and an intracellular kinase domain with serine/threonine kinase activity.^43^ In contrast to TβRI and TβRII, membrane-anchored TβRIII is not a typical receptor that can transduce extracellular signals because it lacks of a domain with serine/threonine activity. It acts primarily as a coreceptor to regulate TGF-β access to TβRI and TβRII.^44^ The type of TGF-β ligand and TβRIII molecule determines binding affinity. β-Glycans, well-characterized members of the TβRIII subfamily, show affinity for all three TGF-β isoforms, with the highest affinity for TGF-β2.^45^ Endoglin, another member of the TβRIII subfamily, selectively binds TGF-β1 and TGF-β3.^44^

Signal transmission efficiency depends on the abundance and availability of ligands and their inhibitors, the number of receptors on the cell surface, and the regulation of kinase activity. TGF-β3-induced signaling pathways are primarily associated with TβRI and TβRII. Once active TGF-β3 is released from an LLC protein, it interacts with a heterotetrameric receptor complex carrying two TβRI subunits and two TβRII subunits.^46^ TβRII proteins are first activated in response to the binding of TGF-β3; then, constitutively active TβRII proteins phosphorylate the juxtamembrane regions of the cytoplasmic domains of TβRI and transmit the signal further into the intracellular space. The heterotetrameric TβRI–TβRII complex is essential for the initiation of TGF-β3 signaling.^47^ A previous study analyzed a synthetic TGF-β3 dimer composed of a wild-type protomer and a receptor-binding-deficient mutant to demonstrate that each pair of TβRI–TβRII heterodimers was necessary for signaling.^46^ TβRII can bind TGF-β ligands directly, while TβRI cannot bind ligands but forms a complex with TβRII in the presence of TGF-β ligands. Moreover, the direct association between TGF-β ligands and TβRII molecules differs among TGF-β isoforms. TGF-β1 and TGF-β3 bind to TβRII even without TβRI. TGF-β2 interacts very weakly with TβRII, which depends on TβRI or TβRIII.^48^

## TGF-β3 signaling

TGF-β3 participates in physiological and pathological functions by elaborately regulating cell metabolism in all tissues of the human body. Intracellular signaling pathways are initiated when ligands activate heterotetrameric TβRI-TβRII complexes. TGF-β3 activates R-Smad-dependent signaling pathways, the canonical pathway of TGF-β family members, and R-Smad-independent signaling pathways.

### The R-Smad-dependent TGF-β3 signaling pathway and its role in cartilage

Smad proteins are canonical downstream mediators of TGF-β3-induced signaling pathways. After activation of TβRs induced by TGF-β3, intracellular Smad proteins transduce signals from the plasma membrane to the nucleus, ultimately eliciting transcriptional responses of target genes.^49^ To date, three types of Smads have been identified: including R-Smads (Smad1, Smad2, Smad3, Smad5 and Smad8/9), a co-Smad (Smad4), and I-Smads (Smad6 and Smad7).

R-Smads and the co-Smad have a unique structure consisting of highly conserved Mad homology 1 (MH1) in the N-terminus and Mad homology 2 (MH2) in the C-terminus end connected via a proline-rich linker region.^50^ The MH1 domain is critical for regulating transcriptional activity by binding with DNA, and the MH2 domain interacts with other proteins and directs the oligomerization of Smad proteins.^51^ Some cofactors that influence gene expression also function by binding to the MH2 domain. R-Smads are further classified into two groups on the basis of structures and functions. One group comprises Smad2 and Smad3, and another group comprises Smad1, Smad5 and Smad8/9. The two different groups of R-Smads direct the behaviors of chondrogenic and osteogenic cells antagonistically by activating and/or blocking the transcription of Runt-related transcription factor 2 (Runx2).^52^ In addition, Smad2/3 can be phosphorylated by TGF-β isoforms, Nodal, and activin. Smad1, 5 and 8 are activated by BMP signaling.^15^ These two different Smad groups are activated by different TβRI proteins and bind to different DNA sequences. Specifically, ALK1/2/3/6 proteins phosphorylate Smad1/5/9, and Smad1/5/9/4 complexes bind to the GCCGCGC sequence. ALK4/5/7 proteins phosphorylate Smad2/3, and Smad2/3/4 complexes, which bind to the GTCTAGAC sequence.^53^ I-Smad proteins inhibit TGF-β3-triggered R-Smad signaling pathways by directly binding to R-Smads and blocking their modification, including phosphorylation and threonylation. Smad6 generally participates in the inhibition of Smad1/5/9, and Smad7 always inactivates Smad2/3.^54^ The I-Smad proteins play inhibitory roles via three mechanisms: dephosphorylation and degradation of TβRs to inhibit the activation of R-Smads, inhibition of the association between R-Smads and specific DNA motifs, and inhibition of the formation of R-Smad/co-Smad complexes.^50^

**Fig. 2:**
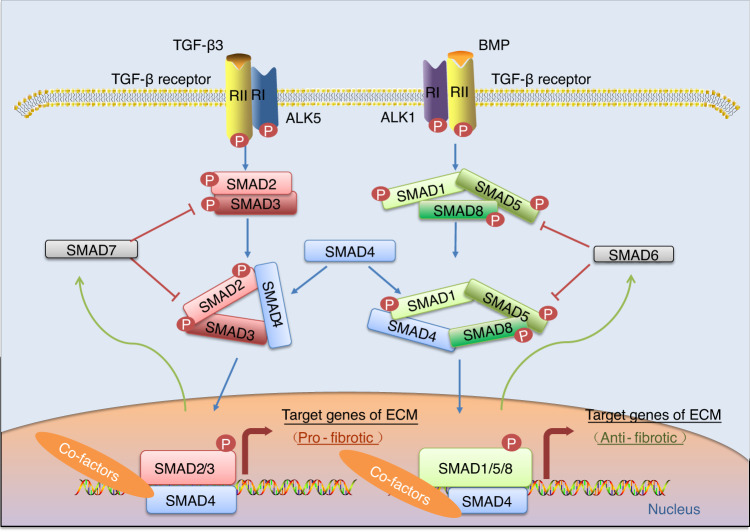
Schematic diagram showing the transforming growth factor-beta 3 (TGF-β3)-mediated R-Smad-dependent signaling pathway. Smad proteins transduce signals from the cell membrane to the nucleus and regulate gene expression after TGF-β3 binding to a TGF-β receptor (TβR). TGF-β3 plays a dual role in articular cartilage. It is involved in cartilaginous homeostasis via the classical Smad2/3 pathway and chondrocyte hypertrophy via the Smad1/5/8 pathway. The partner common to both pathways, Smad4, and inhibitory Smads (Smad6 and Smad7) play important roles. BMP bone morphogenetic protein, ALK1 activin receptor-like-kinase 1, ALK5 activin receptor-like kinase 5

Similar to signal transduction mediated by TGF-β1, TGF-β3 can drive the R-Smad-dependent signaling pathway, as demonstrated by several reports (Fig. 2). Activated TβRI typically induces the phosphorylation of the C-terminal Ser-Ser-X-Ser (SSXS) sequence (a motif of the MH2 domain) downstream of R-Smad proteins after the formation of the heterotetrameric complex. Then, co-Smad is recruited and facilitates the formation of a heterotrimer consisting of two phosphorylated R-Smads (Smad2 and Smad3) and the co-Smad.^55^ The complex enters the nucleus to bind with a DNA strand via the MH1 domain, which carries a nuclear localization signal, and influences gene expression.^56^ Whereas the Smad complex alone interacts weakly with DNA, the affinity is enhanced by transcription factors and cofactors. In collaboration with other nuclear cofactor proteins, the R-Smad/co-Smad complexes regulate gene transcription.^57^ These transcriptional cofactors, whether coactivators or corepressors, are regulated by other signaling pathways that participate in modifying initial Smad signaling, thereby allowing pleiotropic responses to TGF-β3. In cartilage, TGF-β3 increases the production of type II collagen and decreases the production of type I collagen via Smad2/3-mediated signaling. TGF-β3-induced R-Smad-dependent signaling pathways has been confirmed to collaborate with the transcription factor SRY‑box transcription factor 9 (SOX9) in modulating the gene expression of chondrocyte-specific markers, including type II collagen and aggrecan.^56^ Further studies have indicated that SOX9-dependent chondrogenesis are promoted by an increased association between SOX9 and Smad3/4. Moreover, transcription coactivators/factors, such as p300, FoxO (FoxO1, 3, and 4), Sp1, and cJun/c-Fos, facilitate the formation and stabilization of the SOX9–Smad3/4 complex.^58,59^

### The noncanonical TGF-β3 signaling pathway and its role in cartilage

**Fig. 3:**
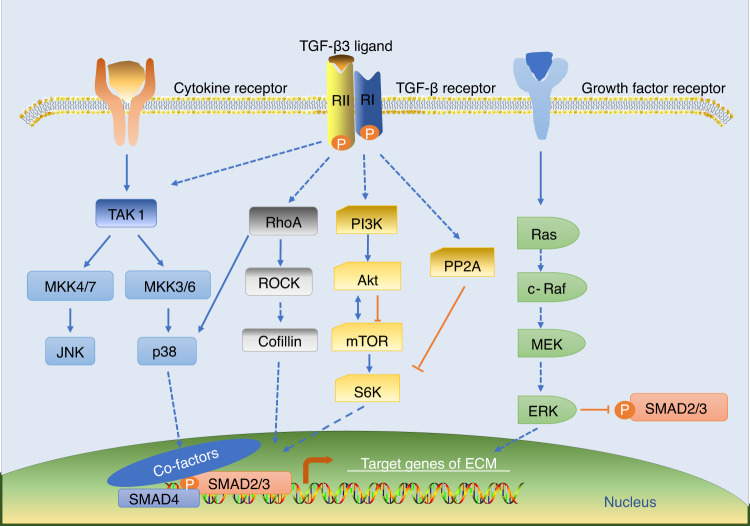
Schematic diagram showing the noncanonical transforming growth factor-beta 3 (TGF-β3) signaling pathway. TGF-β3 activates R-Smad-independent pathways mediated by mitogen-activated protein kinases (MAPKs) (including extracellular regulated kinase (ERK), p38 kinase, and c-Jun N-terminal kinase (JNK)), phosphoinositide 3-kinase (PI3K)/protein kinase B (AKT)/mammalian target of rapamycin (mTOR), and Rho-like GTPase (Ras, RhoA, Rac1, CDC42). These pathways function independently but interact with each other to regulate cartilage development and maintenance. TAK1 transforming growth factor-β-activated kinase 1, MKK3/4/6/7 mitogen-activated protein kinase 3/4/6/7, ROCK Rho-associated protein kinase, S6K S6 kinase, PP2A protein phosphatase 2A, MEK mitogen-activated protein kinase

TGF-β3 activates the noncanonical pathway (the R-Smad-independent pathway) mediated by MAPKs (ERK1/2, JNK, p38), PI3K/AKT/mTOR, and Rho-like GTPase (Ras, RhoA, Rac1, CDC42) (Fig. 3). TGF-β3-induced microRNAs (miRNAs), such as miRNA-410 and miRNA-495, also regulate cellular metabolism at the posttranscriptional level.^48,60^

The MAPK family primarily include ERKs (ERK1 and ERK2), JNKs (JNK1, JNK2, and JNK3), p38 isoforms (p38α, p38β, p38γ, and p38δ) and a fourth MAPK, ERK5. Each MAPK family member is activated by a specific upstream MAPK kinase (MKK), which is highly selective in phosphorylating a specific MAPK. For instance, ERK1 is the phosphorylation substrate of MKK1, and JNK1/ERK5/p38 can be selectively phosphorylated by MKK4/MKK5/MKK6.^61^ Before phosphorylating MAPK, TβRs first activates MAPK kinase kinase and MKK.^62^ TGF-β3-mediated MAPK pathways play significant roles in the regulation of chondrocyte behaviors, including their proliferation, maturation, differentiation, and death.^63^ Different MAPK members play disparate roles in regulating chondrocyte metabolism. For example, p38 and ERK play essential but opposing roles in chondrogenesis, but the effect that JNK exerts on chondrogenic differentiation is less profound than that of p38 and ERK.^64^ During chondrogenesis, the phosphorylation of p38 is enhanced, which is consistent with the increase in Smad2 phosphorylation. In contrast, the phosphorylation of ERK is rapid, transient, and diminished. Therefore, p38 is necessary for chondrogenesis and is slow acting, playing a prolonged role in the process, and ERK signaling may repress chondrogenesis.^65^ Another study pointed out that p38 promoted chondrogenesis by stabilizing SOX9 mRNA in chondrocytes.^66^ However, there are conflicting reports about the effect of p38 on the chondrocyte phenotype. Previous studies have suggested that p38 is involved in chondrocyte phenotype maintenance.^67,68^ Another report put forward a different viewpoint, suggesting that p38 signaling is associated with primary chondrocyte dedifferentiation.^69^ To date, the precise role of p38 in chondrocyte phenotype acquisition remains is unclear. In addition to regulating chondrogenesis and chondrocyte phenotype, p38 plays roles in stress- and inflammation-related activity in chondrocytes.^70^ Transforming growth factor-β-activated protein kinase 1 (TAK1), a signaling molecule upstream of p38, also plays important roles in cartilage physiology.^71^ TAK1 promotes the expression of Type II collagen in chondrocytes by upregulating SOX9 expression.^72^ Studies on mice have shown that the absence of TAK1 affects the proliferation and maturation of chondrocytes, leading to severe cartilage diseases and death.^73^ Similarly, the role of ERK is intricate and hinges on various intracellular and extracellular conditions. Multiple studies have reported that ERK plays a dual role by protecting and inhibiting in cartilage growth and development. Even though ERK is an inhibitor of chondrogenesis, ERK plays a positive and protective role in mature chondrocytes. Elevated ERK levels lead to TGF-β3-induced chondrocyte proliferation and the gene expression of aggrecan and Type II collagen.^74^

PI3K/AKT/mTOR and RhoA/Rho-associated protein kinase (ROCK) are also in the R-Smad-independent signaling pathway. mTOR, a factor downstream of PI3K, is associated with chondroblast growth and proliferation. mTOR is upregulated during OA but downregulated during chondroprotective autophagy.^75,76^ Regarding the RhoA/ROCK pathway, previous studies have found that RhoA-activated ROCK inhibits the mRNA expression of SOX9, Type II collagen, and aggrecan in primary chondrocytes.^77^ In contrast to the results of those on two-dimensional (2D) in vitro cultures, studies based on three-dimensional (3D) micromass cultures have shown that ROCK inhibition increased SOX9 mRNA expression but decreased the expression of SOX9-targeted aggrecan, leading to the conclusion that RhoA/ROCK regulates SOX9 and cartilage ECM proteins in a context-dependent manner.^78^ Another study reported that TGF-β treatment resulted in a ROCK-dependent increase in the phosphorylation and nuclear accumulation of SOX9, and this increase was achieved via the direct interaction between ROCK and SOX9.^79^

Taken together, the data suggest that TGF-β3 can activate a variety of noncanonical intracellular signaling pathways independent of Smads. These Smad-independent signaling molecules interact with the canonical TGF-β3 signaling pathway in a cell-type-specific manner, contributing to the formation of a complex network of signaling molecules engaged in crosstalk. In cartilaginous cells, the interaction between Smads and MAPKs is common and complicated. TGF-β3-induced MAPK activation may result from cellular responses triggered by Smad-dependent transcription. On the other hand, activated MAPKs are involved in Smad-mediated transcriptional regulation by directly phosphorylating Smad proteins and/or influencing their downstream molecules.^80^ For example, TGF-β3 increased the expression of chondroitin sulfate synthase 1 (CHSY1), an enzyme indispensable for the synthesis of proteoglycans, through the activation of Smad2/3 in rat nucleus pulposus cells. Moreover, the three major MAPK members, namely, ERK, p38 and JNK, were all markedly stimulated upon TGF-β3 treatment. Subsequently, MAPK-activated AP1 and Sp1 transcription factors selectively interacted with the nuclear translocated Smad2 and facilitated the transcriptional activation of CHSY1.^81^

MiRNAs are noncoding RNAs 18–24 nucleotides in length. They can recognize and bind specific sequences of the 3ʹ-untranslated region (3ʹ-UTR) of a target mRNA.^82^ TGF-β3-induced signaling influences the expression of numerous miRNAs, which can also mediate crosstalk between TGF-β3 signaling and other pathways, collectively directing chondrogenesis and chondrogenic differentiation (Table 1).^83^ For example, elevated miRNA-410 participates in the TGF-β3-induced chondrogenic differentiation of mesenchymal stem cells (MSCs) by binding to the 3ʹ-UTR of the Wnt3a gene, thus regulating the Wnt signaling pathway and increasing the expression of multiple chondrogenic markers, including Type II collagen, SOX9, aggrecan, and hyaluronan synthase 2.^84^ In contrast, miRNA-495 plays opposite roles in chondrogenic differentiation by directly binding to the SOX9 gene and downregulating the expression of Type II collagen and aggrecan.^85^

**Table 1:** TGF-β3-induced microRNAs that regulate chondrogenic differentiation

MicroRNA	Response to TGF-β3	Mechanism of action	Research objects	Ref.
MicroRNA-199b-5p	↑	Promotes chondrocyte differentiation by targeting JAG1 and upregulating chondrogenic markers (SOX9, aggrecan, and Type II collagen)	Human BMSCs and the C3H10T1/2 cell line	^83^
MicroRNA-410	↑	Promotes chondrocyte differentiation by targeting Wnt3a and upregulating chondrogenic markers (Type II collagen, SOX9, aggrecan, and hyaluronan synthase 2)	Human BMSCs	^84^
MicroRNA-495	↓	Inhibits chondrocyte differentiation by targeting SOX9 and downregulating Type II collagen and aggrecan	Human BMSCs	^85^
MicroRNA-488	↑	Promotes chondrocyte differentiation by targeting ZIP-8 and inhibiting MMP-13 activity	Human articular chondrocytes	^193^
MicroRNA-181b	↓	Inhibits chondrocyte differentiation by upregulating MMPs and possibly by accelerating cartilage ECM degradation	Chick wing limb bud mesenchymal cells	^194^
MicroRNA-145	↓	Inhibits chondrocyte differentiation by targeting SOX9 and downregulating chondrogenic markers (Type II collagen, aggrecan, cartilage oligomeric matrix protein, Type IX collagen, and Type XI collagen)	Murine BMSCs	^195^

## The basal role of TGF-β3 in the life cycle of chondrocytes

**Fig. 4:**
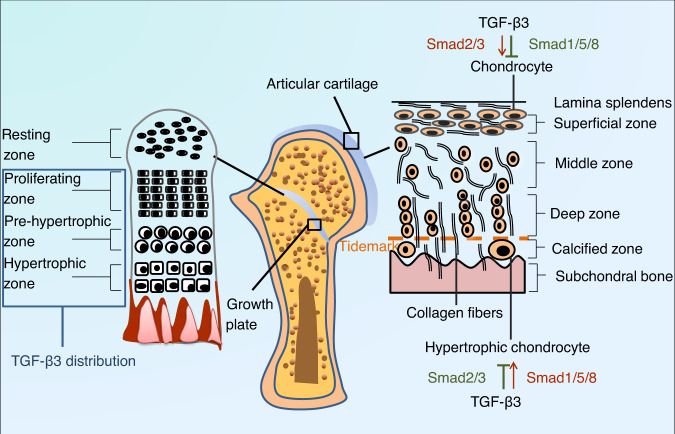
Schematic diagram showing the basic structure of cartilage and the distribution of transforming growth factor-beta 3 (TGF-β3). Chondrocytes are divided into articular chondrocytes located in articular cartilage and growth plate chondrocytes residing in growth plate cartilage based on the degree of their terminal differentiation. Growth plate cartilage comprises the resting zone, proliferative zone, pre-hypertrophic zone and hypertrophic zone. TGF-β3 is mainly expressed in chondrocytes located in the proliferative and hypertrophic zones. Articular cartilage consists of five distinct zones: the lamina splendens, superficial zone, middle zone, deep zone and calcified zone. TGF-β3 is involved in chondrogenic differentiation and chondrocyte hypertrophy through the Smad2/3 pathway and Smad1/5/8 pathway, which play antagonistic roles during different stages of cartilage differentiation

Chondrocytes are classified into articular chondrocytes located in articular cartilage and growth plate chondrocytes residing in growth plate cartilage based on the degree of terminal differentiation (Fig. 4).

The lubricated surface of articular cartilage is reversibly deformed and moves with minimal friction. The histological structure of cartilage allows it to disseminate shear, tensile, and compressive forces.^86^ Based on structural differences, cartilage can be divided into five unique zones: the lamina splendens, superficial zone, middle zone, deep zone, and calcified zone.^87^ Each zone exhibits its own histological and functional features. At the surface of articular cartilage, the lamina splendens comprise a cell-free layer. All collagen fibers in this zone are oriented parallel to the surface, which enables it to resist pressure. In addition, it contains a large amount of HA and proteoglycan 4 (PRG4), maintaining low friction and boundary lubrication.^88^ Below the superficial zone is underneath the lamina splendens, and it also contains high levels of HA and PRG4, with tightly packed collagen fibrils oriented parallel to the surface. In contrast to lamina splendens, however, the superficial zone contains a relatively high number of cells, which have a flattened morphology and are oriented parallel to the surface.^89^ The middle zone is underneath the superficial zone and accounts for 40%–60% of the total cartilage volume and comprises spherical and rare chondrocytes. Collagen fibers are oriented in oblique orientation to the cartilage surface, endowing this zone with low stress tolerance. Functionally, the middle zone is considered the first layer that resists compressive forces.^87^ A tidemark distinguishes the two basal zones: the deep zone and the calcified zone. Chondrocytes in the deep zone proliferate unidirectionally and are organized in columns oriented vertical to the cartilage surface and parallel to the collagen fibrils. This zone exhibits the highest proteoglycan level and is critical for providing the greatest resistance to compressive forces.^90^ The calcified zone, underneath the tidemark, forms the interface between bone and cartilage by anchoring the collagen fibrils in the deep zone to subchondral bone. This zone comprises hypertrophic-like chondrocytes undergoing endochondral ossification.^91^

Growth plate cartilage comprises the resting zone, proliferative zone, pre-hypertrophic zone, and hypertrophic zone.^12,90,92^ Cells in the resting zone are small, uniform, compact and chondrocytes critical for protein synthesis and exist as individual cells or in pairs. In the proliferative zone, flat chondrocytes are clearly divided into longitudinal columns and rapidly duplicate. A true germinal layer is found only at the base of columns and produces Type II and Type XI collagen.^93^ Underneath the proliferative zone, in the pre-hypertrophic zone and hypertrophic zone, chondrocytes terminally differentiate into pre-hypertrophic chondrocytes and hypertrophic chondrocytes, respectively, and exhibit a relatively large and swollen morphology.^94^ Once terminal differentiation is initiated, mature chondrocytes produce vascular endothelial growth factor to induce blood vessel invasion and matrix metalloproteinases (MMPs) to promote the degradation of the cartilage ECM by chondroclasts, contributing to the formation of a primary ossification center.^95^ Growth plate chondrocytes eventually undergo apoptosis and are replaced by bone cells during endochondral ossification. Correspondingly, the cartilage matrix is gradually converted into bone matrix.^96,97^

Chondrocytes exhibit a life cycle of proliferation, differentiation, maturation, and apoptosis.^98^ TGF-β3 plays crucial regulatory roles in these processes by interacting directly with TβRs on the outer cell membrane and triggering a cascade of molecular alterations involving SOX9. Then, the expression of multiple cartilage ECM proteins, such as Type II collagen and aggrecan, is further regulated.^17^ TGF-β3 is expressed in chondrocytes located in proliferative and hypertrophic zones. Notably, TGF-β3 is widely distributed at sites of intramembranous ossification.^99^ TβRI and TβRII, the two TβRs for which TGF-β3 shows strong affinity, are distributed in the hypertrophic zone and the calcified zone of the neonatal rib as well as in the resting zone and proliferative zone of a developing osteophyte.^100^ The variance in its distribution indicates that TGF-β3 plays a dual function.

### Chondrocyte proliferation and migration

The limited repair capacity of cartilage is directly related to chondrocyte proliferation and migration into a wound site. In addition to its association with cartilage repair and regeneration, cell proliferation is important for the growth and development of cartilage. Therefore, evaluating the role played by TGF-β3 in chondrocyte proliferation may help us optimize a treatment strategy for cartilage defects. Sefat et al. reported that the proliferation rate of primary chondrocytes decreased, with more cells presenting with a fibroblastic morphology after treatment with TGF-β3.^101^ These variations were caused by the positive effect of TGF-β3 on chondrocyte dedifferentiation, a rapid change in phenotype and gene expression generally observed in vitro, particularly cultured chondrocyte monolayers. The inhibitory role played by TGF-β3 on cell proliferation has also been verified during the chondrogenesis of chick leg bud mesenchymal cells.^102^ However, Martinez-Alvernia et al. put forward a contradictory opinion by suggesting that exogenous TGF-β3 increases chondrocyte proliferation.^103^ James et al. also observed that TGF-β3 significantly increased cell proliferation in posterior frontal suture-derived mesenchymal cells.^104^ In addition, cell proliferation was promoted when chondrocytes were cocultured with adipose-derived stem cells (ADSCs) in medium supplemented with TGF-β3.^105^ In summary, the recent research findings are contradictory, and no unanimous conclusions can be drawn.

Nevertheless, TGF-β3 is widely used in cell-based cartilage tissue engineering owing to its other functions, such as the promotion of cell migration. Makhijani et al. reported that all three TGF-β isoforms stimulate chemotaxis in C3H10T1/2 cells.^106^ The migration of OP9 MSCs has also been efficiently induced by TGF-β3.^107^ The chemotactic role of TGF-β3 has been confirmed via in vivo experiments. After humeral head resection, rabbits were implanted with TGF-β3-infused bioscaffolds or TGF-β3-free bioscaffolds. Then, the regenerated cartilage samples were collected and analyzed, and the results revealed that compared with the number of cells that spontaneously migrated without TGF-β3 stimulation, ~130% more cells were recruited to the regenerated cartilage in the rabbits treated with TGF-β3.^108^ Brittberg et al. found that a suitable dose of TGF-β3 induced the migration of MSCs to cartilage lesions and the formation of hyaline-like grafts.^109^ With advancements in tissue engineering and regenerative medicine, some cofactors have also been applied, such as BMPs and dexamethasone, which play synergistic roles.^110,111^

### Chondrocyte differentiation

Chondrogenic differentiation, a prerequisite for cartilage formation and endochondral ossification, begins with cell proliferation, precartilage condensation, and mesenchymal cell differentiation into chondrocytes, contributing to the formation of a cartilaginous template for future bone development.^112,113^ The cartilaginous template undergoes cell proliferation, hypertrophic differentiation, calcification, and apoptosis and is eventually replaced by bone.

#### Chondrogenic differentiation

MSCs residing in the bone marrow of adult humans retain the capacity to proliferate and differentiate into multiple cell lineages, including chondrocytes. Generally, the role played by TGF-β3 in chondrogenesis is cell type specific. Through the activation of Notch signaling, TGF-β3 plays an inhibitory role on chondrocytes in limb bud mesenchymal cells by repressing their proliferation and precartilage condensation.^102^ Similarly, TGF-β3 treatment inhibits chondrogenesis of chick leg bud mesenchymal cells by downregulating connexin 43 and integrin beta4 mediated by the suppression of protein kinase C-alpha (PKC-α) and the activation of ERK.^114^ Jin et al. demonstrated that TGF-β3 stimulated chondrogenic differentiation of wing bud mesenchymal cells through the activation of the PKC-α and p38 MAPK pathways.^115^ They hypothesized that the disparity is attributable to the multiple functions of TGF-β3. Specifically, TGF-β3 showed stimulated chick wing chondroblast differentiation into chondrocytes and inhibitory effects on chick leg chondroblast differentiation, thus establishing fore- and hind-limb identity.^114^

Despite its mixed inductive and inhibitory effects, TGF-β3 is considered to induce greater chondrogenic effects than TGF-β1, but TGF-β3 is not sufficient for promoting chondrogenic differentiation in monolayers.^116,117^ James et al. explained the different effects of TGF-β1 and TGF-β3 on chondrogenic differentiation in posterior frontal suture-derived mesenchymal cells.^104^ Notably, TGF-β1 promotes precartilage condensation and chondrocyte differentiation, while TGF-β3 may predominantly induce chondrogenesis via the significant expansion of chondroprogenitor cells. Currently, TGF-β3 is used as a standard additive that is encapsulated in a variety of scaffolds to induce the chondrogenesis of MSCs.^118^ The successfulness of this application has been supported by numerous in vitro, animal and human in vivo studies. For example, chondrogenic differentiation of human MSCs was induced in pellet culture after the medium was supplemented with dexamethasone, bone morphogenetic protein 6 (BMP6) and TGF-β3. This optimized medium enhanced the weight of the cartilage pellets approximately tenfold. In addition, the production of proteoglycans and the gene expression of Type II procollagen and Type X collagen were both increased.^119^ Sasaki et al. developed a model of meniscus radial tears and found that injection of ADSCs encapsulated in a TGF-β3-preloaded photo-cross-linkable hydrogel stimulated robust matrix-sulfated proteoglycan deposition and enhanced healing of meniscal radial tears. This outcome shows a novel strategy to repair meniscal radial tears and minimize further osteoarthritic joint degeneration.^120^ Similar results were reported for another in vitro experiment in which human MSCs seeded in silk-elastin-like polymer (SELP) scaffolds containing TGF-β3 promoted significant increases in glycosaminoglycan and total collagen content.^121^ TGF-β3 may also be delivered via direct encapsulation within HA hydrogels. TGF-β3 retained within a hydrogel altered chondrogenic gene expression of encapsulated MSCs when implanted in mice.^122^

#### Terminal hypertrophic differentiation

Most of the skeleton, such as the long bones in the limbs and vertebral columns, is formed through endochondral ossification.^96^ The condensation of MSCs is a prerequisite for endochondral bone formation. MSCs differentiate into chondrocytes, which are characterized by the production and accumulation of cartilage-specific proteins, such as Type II collagen and aggrecan. Apparently, unrestricted differentiation of precursor cells into the chondrocyte lineage leads to the formation of bones but not permanent cartilage, which develops only when this route is blocked in articular cartilage.^91^ In contrast to permanent cartilage, the temporary hyaline cartilage-like growth plate is ultimately replaced by bone. In growth plates, maturing chondrocytes produce a cartilaginous matrix and then exit the cell cycle, undergo hypertrophic differentiation, produce Type X collagen, and show alkaline phosphatase activity.^123^ Once hypertrophic chondrocytes have terminally differentiated, vascularization and focal calcification are initiated; finally, the cartilaginous matrix is mineralized, and the cells undergo apoptosis.^95,124^ Terminal hypertrophic differentiation is mainly controlled by the two transcription factors SOX9 and Runx2. SOX9 is a negative regulator of hypertrophic differentiation, cartilage angiogenesis, and bone marrow formation.^125^ Runx2 is a positive regulator of hypertrophic differentiation and cartilage vascularization.

The role for TGF-β3 in chondrocyte hypertrophic differentiation has been reported. Initial research revealed that the terminal differentiation of cranial chondrocytes was arrested by TGF-β3 and FGF2, which synergistically suppressed the expression of terminal differentiation markers such as Type X collagen, matrix metalloproteinase 13 (MMP13, also known as collagenase 3) and Runx2.^126^ However, some studies put forward different opinions, suggesting that TGF-β3 upregulates the expression of MMP13, a marker of OA cartilage.^127^ This regulation is mediated by the altered expression of inflammatory cytokines, such as interleukin-1 (IL-1).^128^ In addition, Hennig et al. observed an induced hypertrophic phenotype in ADSCs after successful chondrogenic induction via TGF-β3 and BMP6.^129^ Similar results showing that differentiating MSCs express hypertrophic markers under standard chondrogenic conditions have also been reported by Mueller et al;^116^ this group pointed out that the hypertrophy observed in an in vitro model was most significantly increased after 14 days of chondrogenic predifferentiation. Indeed, TGF-β3 not only acts as a chondro-inductive growth factor but also predisposes MSCs to hypertrophy. The dual role of TGF-β3 in the terminal hypertrophic differentiation of chondrocytes is mediated by the Smad2/3 and Smad1/5/8 pathways. TGF-β3-induced Smad2/3 signaling induced via the activation of ALK5 helps to preserve the phenotype of chondrocytes by inhibiting hypertrophy and terminal differentiation.^130^ In contrast, TGF-β3-induced Smad1/5/8 signaling mediated via the activation of ALK1 facilitates chondrocyte hypertrophy.^131^

### Chondrocyte death

Dynamic and accurate regulation of cell death in chondrocytes is pivotal in skeletal development, maintenance, and repair as well as in the pathological course of OA and osteoporosis (OP).^132,133^ Different types of cell death in cartilage have been reported, including three types of physiological/programmed cell death (apoptosis, chondroptosis, and paraptosis) and one type of pathological cell death (necrosis).^134^ Apoptosis is a highly regulated, “active” cell death (as opposed to “passive”) modality associated with homeostasis and senescence.^135^ It is an enzyme-dependent biochemical process that causes minimal damage to the surrounding microenvironment. The initiation of apoptosis relies on the activation of a series of caspases.^136^ Apoptosis has been reported to be critical to the regulation of cartilage development. Mrugala et al. used DNA microarrays and performed gene expression profiling of human MSCs in the different phases of chondrogenic differentiation induced by TGF-β3/BMP2.^137^ They found that apoptosis-related genes were highly expressed at an early stage, which is characterized by cell attachment, and the later stage, which is characterized by chondrocyte hypertrophy. Dysregulation of apoptosis results in developmental abnormalities, degenerative diseases, and cancer. Increasing evidence has suggested that programmed cell death is an outcome of multiple modalities, not merely apoptosis, and can involve different pathways of active self-destruction, such as those in autophagic cell death.^138^ Autophagy is a catabolic process in all eukaryotic cells that removes waste and damaged cellular components via lysosomal degradation.^139^ Autophagic cell death, which may be an alternative to classical apoptosis (classified as Type I cell death), is classified as Type II cell death.^138^ It is mediated by lysosomal proteinases, especially cathepsins B and D, but not caspases.^140^ Autophagy provides the timely regulation of chondrocyte maturation and ECM formation. Chondrocyte hypertrophy may be promoted in the absence of autophagy.^141^ Necrosis, however, is uncontrolled cell death and usually follows severe damage and subsequently affects tissue surrounding a lesion.^136^

The regulatory role played TGF-β1 in chondrocyte death has been extensively explored. Downstream signaling pathways that govern this process have also been identified.^133^ However, studies on the interaction between TGF-β3 and chondrocyte apoptosis/necrosis/autophagy are rare and primarily phenomenological. Among those who published articles that we retrieved and analyzed, most researchers paid focused on the role played TGF-β3 in chondrocyte apoptosis. Exogenous TGF-β3 has been found to decrease the apoptosis rate during chondrogenic differentiation of chick leg bud mesenchymal cells through the suppression of integrin β4 expression, activation of ERK, and suppression of PKC-α activation.^114^ An in vivo study reported that TGF-β2 and TGF-β3 played roles in tissue shaping during limb formation by regulating apoptosis. The mesenchyme of interdigital spaces shows chondrogenic potential, and interdigital cells need to undergo apoptotic cell death during limb development.^142^ Dünker et al. found that the apoptosis rate in the interdigital spaces of forelimbs and hindlimbs was significantly reduced in TGF-β2^−/−^TGF-β3^−/−^ double-knockout mice as well as in TGF-β2^−/−^TGF-β3^+/−^ mutants. Moreover, large hypertrophied chondrocytes were observed within the interdigital region of the two mutants, which meant chondrogenesis had been accelerated, and an advanced stage of enchondral ossification had been reached; in contrast, wild-type mouse digits contained small proliferating chondrocytes.^143^ Similarly, Cheah et al. knocked down TGF-β3 in zebrafish and found that normal chondrogenesis in the craniofacial region was dependent on the tight regulation of TGF-β3. They proposed that both TGF-β3 suppression and overexpression led to reduced chondrocyte formation but to different degrees and through different mechanisms. TGF-β3 dysregulation-induced apoptosis was detected in both situations and was considered to be the common mediating factor.^144^ In summary, the regulatory networks and mechanisms through which TGF-β3 affects chondrocyte death still need to be elucidated through further study.

## Potential therapeutic roles of TGF-β3 in osteoarthritis (OA)

### Basic characteristics of OA

Of all the joint diseases, OA is the most common degenerative disorder, and it is highly associated with age, exerting extensive influence on an individual physical health, leading to disability, and on the health care system and society.^11^ Debilitating and chronic joint disorders mainly affect weight-bearing joints, such as hips and knee.^145^ Etiological factors, such as abnormal joint loading, degenerative aging, and genetic predisposition, may irreversibly contribute to cartilage degradation.^146^ To date, effective reparative approaches to these conditions have not been established, as the precise pathogenesis of OA remains unclear.^12,86,147^ The current pathophysiological concept suggests that OA as a degenerative disease of the whole joint and that all components of the diarthrodial joint involving the synovium, subchondral bone and cartilage contribute to pathologic abnormalities.^148^ More specifically, osteophyte formation, subchondral bone sclerosis underneath eroded cartilage, and disruption of the tidemark, which is accompanied by angiogenesis at the osteochondral junction, are the characteristics of OA. In addition, ossification at the sites of ligament, tendon, and joint capsule insertion into bone is also observed, ultimately leading to joint asymmetry and malalignment.^149,150^

Cartilage degeneration, characterized by progressive loss of aggrecan and Type II collagen, had been previously considered to be the central pathological feature of OA. Elevated expression levels of multiple MMPs, such as MMP2, MMP9, and MMP13, are involved in this degenerative process.^151^ In recent years, however, alterations in the underlying subchondral bone have garnered increasing interest. Immoderate bone proliferation contributes to an eburnated subchondral plate and a profound increase in its thickness, increasing stiffness, altering the mechanical property of subchondral bone and changing the distribution of articular cartilage stress.^152^ The synovium is also involved in several stages of this pathological process. In the early phase of OA, catabolic cytokines released from the synovium induce transient chondrocyte proliferation as well as increased synthesis of type II collagen and aggrecan in an attempt to initiate repair.^153^ Subsequent synovial inflammation, commonly attributed to abnormal molecular signaling in damaged cartilage, leads to the constant production of multiple MMPs and ultimately to greater cartilage destruction.^154^

### Involvement of TGF-β3 in the pathology of OA

**Fig. 5:**
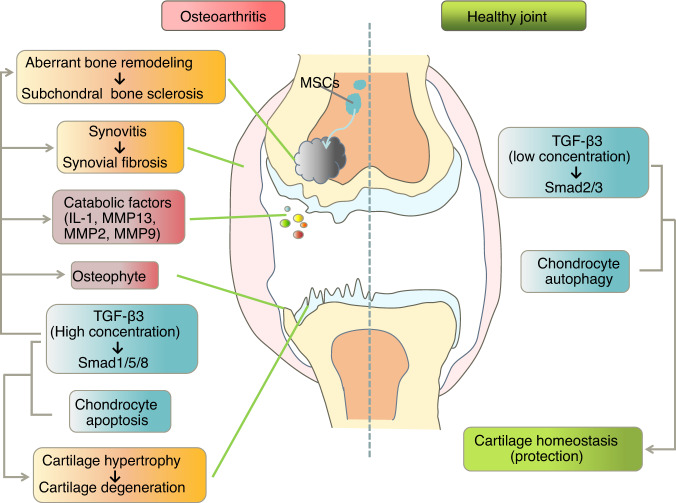
Schematic diagram showing the role of transforming growth factor-beta 3 (TGF-β3) in osteoarthritic and healthy joints. In healthy articular cartilage, low concentrations of TGF-β3 promote metabolic balance through the Smad2/3 pathway. Chondrocyte autophagy also plays a protective role. TGF-β3 is also involved in many aspects of osteoarthritis (OA) pathology mediated through the Smad1/5/8 pathway. High concentrations of TGF-β3 in osteoarthritic joints induce the production of catabolic factors and chondrocyte hypertrophy, ultimately resulting in cartilage matrix degradation, osteophyte formation, and synovial fibrosis. In addition, high levels of TGF-β3 upregulate the expression of Runt-related transcription factor 2 (Runx2) via the Smad1/5/8 pathway, leading to aberrant bone remodeling and further subchondral bone sclerosis. In addition, chondrocyte switching from autophagy to apoptosis has been implicated in OA progression. IL-1 interleukin-1, MMP13 matrix metalloproteinase 13, MMP2 matrix metalloproteinase 2, MMP9 matrix metalloproteinase 9

The involvement of TGF‑β3 in OA has been confirmed with human pathological clinical data. Kapetanakis et al. reported that the serum protein level of TGF-β3 was markedly higher in patients with knee OA than in controls, as determined via enzyme-linked immunosorbent assay. This increase in TGF-β3 was strongly correlated with pain, functionality, and radiographic staging in OA.^155^ To adequately evaluate and refine joint lesion characterization, a better understanding of the pathophysiologic mechanisms is necessary. For that purpose, we focus on the role of TGF-β3 in the context of OA pathology (Fig. 5).

TGF-β3-mediated Smad2/3 signaling plays antihypertrophic and anti‑inflammatory roles in young and healthy cartilage. In contrast, TGF-β3-induced Smad1/5/8 signaling is associated with pro-hypertrophic control in pathological cartilage.^131,156^ The activation of these two differential pathways hinges on the concentration of active TGF‑β3. A high concentration of TGF-β3 preferentially stimulates Smad1/5/8 signaling, while a low concentration predominantly stimulates Smad2/3 signaling.^157^ In healthy joints, low levels of active TGF‑β3 stimulate chondrocyte proliferation and induce the deposition of Type II collagen and aggrecan via the Smad2/3 signaling pathway.^158^ This pathway is important in the regulation of joint homeostasis and counteracts pathological chondrocyte hypertrophy in OA, thereby preserving cartilage integrity.^159^ High levels of TGF-β3 in OA joints upregulate the expression of Runx2 via the Smad1/5/8 pathway, which induces further production of MMP13 and leads to chondrocyte hypertrophy, ultimately resulting in ECM degradation, osteophyte formation, synovial fibrosis and chondrocyte apoptosis.^160^ The interaction between the TGF-β3 signaling pathway and catabolic cytokines contributes to in OA cartilage pathology. For example, IL-1β, one of the most potent cytokines triggering an irreversibly destructive cascade of articular cartilage in OA, may inhibit Smad2/3 signaling by downregulating TβRII expression and upregulating Smad7 expression.^161^

Increasing evidence shows an association between OA-induced cartilage degradation and chondrocyte death.^162^ Hwang et al. suggested that chondrocyte death and matrix degradation may form a vicious cycle, with the progression of one aggravating the other.^163^ Among the multiple types of cell death, apoptotic chondrocyte death is drawing considerable attention from researchers exploring the pathogenesis of OA. There is a clear correlation between the degree of cartilage degradation and the chondrocyte apoptosis rate.^164^ Whether chondrocyte apoptosis leads to OA or cartilage degeneration leads to chondrocyte apoptosis has been widely debated, and the cause-and-effect relationship between OA and chondrocyte apoptosis has been difficult to clarify. Autophagy, leading to another form of chondrocyte death, is changed in OA. A switch from chondrocyte autophagy to apoptosis has been implicated in OA progression. Since autophagy inhibits inflammation and reduces chondrocyte apoptosis in OA, promoting chondrocyte autophagy and inhibiting apoptosis may become a therapeutic strategy to cure OA.^165^ In summary, we have mentioned explained that TGF-β3 is involved in the regulation of chondrocyte apoptosis and that both TGF-β3 and chondrocyte death are associated with OA pathogenesis. Therefore, it is reasonable to consider that there might be a relationship between TGF-β3 and chondrocyte death in OA, but we did not find an article discussing this possible interaction.

### The application of TGF-β3 in cartilage tissue engineering

Conventional treatments for OA only relieve symptoms and do not prevent or cure the disease. Nonsurgical treatments include physical therapy and CS supplementation. Some surgical treatments, such as abrasive methods (microfracturing by drilling small holes), are expected to lead to partial recovery of the articular cartilage structure through the migration of progenitor cells derived from the bone marrow to the lesion site following subchondral fracturing.^166^ Although it shows some shortcomings, the technology is used because it works quickly. Autologous chondrocyte implantation (ACI) is an alternative treatment that involves the collection, cultivation, and expansion of autologous chondrocytes. Preliminary cell modification has optimized the technology, but the long-term effects of this treatment method remain controversial.^167^ With the development of tissue engineering and regenerative medicine, the era of cell-based tissue engineered constructs (CTECs) used to repair cartilage damage has been initiated. Pretreated MSCs are placed on a biodegradable scaffold, and growth factors are added before implantation into defective cartilage.^168^ The primary goal of CTEC is to obtain cells in culture that can proliferate and form transparent grafts without causing degradation. This technique significantly reduces the early migration of cells from the defect site because biodegradable scaffolds protect cells from heavy mechanical loads. In addition, the 3D scaffold prolongs the release time of the chondrogenesis activator by ensuring a uniform distribution of stem cells throughout the scaffold.^169^ Some growth factors that regulate chondrogenesis and help to restore damaged cartilage surfaces via gene modulation have recently attracted attention; one of these factors is TGF-β3. Herein, we summarize thirteen papers to demonstrate the application of TGF-β3 using different delivery scaffolds in cartilage tissue engineering (Table 2).

**Table 2:** Selected bibliography demonstrating the application of TGF-β3 in cartilage tissue engineering

Scaffolding materials	Chondrogenesis activators	Cell sources	Results	Conclusions	Ref.
SELP-47K hydrogel scaffold	TGF-β3	Human BMSCs	1. Human MSCs seeded in SELP-47K scaffolds acquired a rounded morphology after stimulation by TGF-β3 2. The MSCs/SELP-47 K constructs exhibited a significant increase in the expression of chondrogenic markers (aggrecan, Type II and X collagen, and SOX9) and the content of cartilage-like matrix (sulfated glycosaminoglycan and total collagen) with the addition of TGF-β3.	SELP-47K hydrogel scaffolds induced cartilage matrix accumulation and in vitro chondrogenic differentiation of human MSCs in the presence of TGF-β3.	^121^
PLGA scaffold	TGF-β3 and BMP7	Human ADSCs	1. Sustained release of BMP7 cooperated with TGF-β3 to promote chondrogenic differentiation 2. MSCs cultured on scaffolds loaded with BMP7 and TGF-β3 exhibited cartilage formation and upregulation of chondrogenic markers (SOX9 and aggrecan).	PLGA scaffolds encapsulating BMP7 and TGF-β3 efficiently delivered a cooperative growth factor combination that induced efficient cartilage formation with human MSCs.	^169^
3D-Bioprinted intervertebral l disc (IVD) scaffold (major components: polycaprolactone, hydrogel, polydopamine nanospheres)	TGF-β3, connective tissue growth factor (CTGF)	Rat BMSCs	1. In vitro studies: controlled-released CTGF and TGF-β3 from an IVD scaffold induced BMSCs differentiation into fibrocartilage and hyaline cartilage-like cells 2. In vivo studies: a reconstructed IVD showed effective biomechanical properties and a zone-specific matrix (glycosaminoglycan and Type II collagen in the core zone, and Type I collagen in the surrounding zone).	3D-Bioprinted IVD scaffolds loaded with CTGF and TGF-β3 showed great potential for clinical application.	^196^
Methacrylated HA (MeHA) scaffold	TGF-β3 and stromal cell-derived factor-1α (SDF-1α**)**	Bovine BMSCs	1. In vitro studies: TGF-β3 enhanced cell proliferation and cartilage matrix formation. Both SDF-1α and TGF-β3 increased cell migration. Dual-factor release (SDF-1α and TGF-β3) from MeHA scaffolds increased cell recruitment and matrix deposition 2. In vivo studies: MeHA scaffolds were used to repair full-thickness cartilage defect in a large animal model. Scaffolds releasing SDF-1α led to inferior healing responses (lower mechanics and lower ICRS II histology score) compared with scaffolds releasing TGF-β3 alone.	1. MeHA scaffolds served as vehicles to delivery growth factors specifically designed to promote cartilage repair in engineered cartilage tissue. 2. Scaffolds releasing only TGF-β3 showed higher chondrogenic capacity than scaffolds releasing SDF-1α alone or scaffolds releasing two factors 3. SDF-1α increased cell penetration ability in vitro, while its local release inhibited neo-cartilage tissue regeneration in vivo.	^197^
3D-Printed temporomandibular joint (TMJ) disc scaffold (major components: polycaprolactone (PCL), PLGA microspheres (µS))	TGF-β3, CTGF	Human BMSCs	1. 3D-printed TMJ disc scaffolds releasing CTGF and TGF-β3 successfully guided MSCs to form native TMJ disc-like fibrocartilage with a heterogeneous matrix (collagen-rich fibrous matrix in the anterior/posterior (AP) bands, fibrocartilaginous matrix in the intermediate zone) 2. A high dose of CTGF/TGF-β3-µS led to significantly higher Type II collagen and aggrecan levels in the intermediate zone, and higher levels of Type I collagen in the AP bands compared with the effect of low-dose µS. 3. A high dose of CTGF/TGF-β3-µS resulted in the formation of fibrocartilage with a coefficient of viscosity that was significantly higher than that in the low-dose and an empty µS. 4. CTGF/TGF-β3 encapsulated PLGA µS induced the formation of fibrocartilage with a significantly lower ratio of relaxation modulus to instantaneous modulus than realized with an empty µS.	1. CTGF/TGF-β3-encapsulated PLGA µS induced fibrocartilage formation in a dose-dependent manner 2. 3D-Printed TMJ disc scaffolds for the spatiotemporal delivery of CTGF and TGF-β3 encapsulated in PLGA µS successfully guided MSCs to form native TMJ disc-like fibrocartilage with anisotropic microstructure, heterogeneous matrix, and region-dependent viscoelastic properties. This 3D-printed scaffold may be a useful tool for regeneration of the TMJ disc.	^198^
Chitosan-agarose (CHAG) scaffold	TGF-β3 and BMP2	Human Wharton’s jelly MSCs (HWJ-MSCs)	1. CHAG scaffolds exhibited a macroporous structure with pore sizes ranging from 75 µm to 300 µm and exhibited a slow degradation rate (18%) 2. HWJ-MSCs seeded on CHAG scaffolds were treated with both BMP2 and TGF-β3, which led to a higher glycosaminoglycan content compared to that in untreated cells or cells treated with either of the growth factor alone.	1. CHAG scaffolds guided HWJ-MSCs to differentiate into the chondrogenic lineage. Additional supplementation of BMP2 and TGF-β3 significantly increased chondrogenesis and neo-ECM synthesis 2. Macroporous CHAG scaffolds can be applied to cartilage repair based on HWJ-MSCs.	^199^
Aqueous-derived silk scaffold	TGF-β3 and dexamethasone	Human BMSCs	1. Chondrogenesis of MSCs in a silk scaffold was evident in evaluations of cartilage-specific ECM gene and protein marker expression 2. After 3 weeks of cultivation with TGF-β3 and dexamethasone, the cell arrangement and Type II collagen distribution of the MSCs/silk constructs were similar to those of native cartilage tissue.	1. Dexamethasone and TGF-β3 were critical for the proliferation and chondrogenesis of MSCs in the silk scaffold 2. The biocompatible and biodegradable 3D silk scaffold provided structural support for MSCs, which formed a dense construct enriched with cartilaginous ECM.	^178^
Silk fibroin (SF) scaffold	TGF-β3 and MGF	Human BMSCs	1. The recruitment of chondrocytes cultured with both MGF and TGF-β3 increased significantly compared with that of cells cultured with TGF-β3 alone in vivo and in vitro 2. MGF increased the production of chondrogenic markers (Type II collagen and aggrecan) and decreased the secretion of type I collagen in TGF-β3 induced MSCs chondrogenesis in vitro 3. Methanol-treated SF scaffolds showed similar compressive modulus to native cartilage ratios, a slow degradation rate, and continuous drug-release curves 4. In vivo studies: after implantation into a defective joint, SF scaffolds showed the best integration into the surrounding tissues, as well as similar architecture and collagen organization to those of native cartilage. TGF-β3- and MGF-based SF scaffolds recruited more CD29+/CD44+ multipotent stem cells and generated a higher level of cartilage-like ECM and lower levels of fibrillar collagens than TGF-β3-immobilized scaffolds.	1. The combination of MGF and TGF-β3 facilitated cartilage regeneration 2. TGF-β3 and MGF dual- functionalized SF scaffolds formed a novel functional scaffold for cartilage repair that inhibited fibrosis enhanced stem cell recruitment and chondrogenic differentiation.	^180^
Alginate bead scaffold, cartilage-derived matrix (CDM) scaffold	TGF-β3 and BMP6	Human ADSCs and human BMSCs	1. Growth factors (TGF-β3 and BMP6) induced chondrocyte phenotype acquisition in both ADSCs and BMSCs in alginate bead scaffolds and in CDM scaffolds 2. BMSCs demonstrated more robust chondrogenesis (indicated by upregulated Type II collagen gene expression and accelerated matrix synthesis) and a greater tendency for acquisition of a hypertrophic phenotype 3. ADSCs showed higher expression of the aggrecan gene in response to BMP6, while BMSCs responded more favorably to TGF-β3.	1. ADSCs and BMSCs are unique cell types with distinct responses to growth factor-induced chondrogenic induction 2. Growth factor-induced chondrogenic differentiation was affected by cell type, specific growth factors, composition of scaffold, and presence of serum 3. CDM scaffolds were novel culture systems for comparing the chondrogenesis of different stem cells under different culture conditions.	^182^
3D-Printed chitosan scaffold	TGF-β3 and BMP6	Human infrapatellar fat pad adipose stem cells (IPFP-ASCs)	1. A cartilage-like matrix formed the top layer of a 3D-printed chitosan scaffold 2. After stimulation by TGF-β3 and BMP6: IPFP-ASCs exhibited a chondrocyte morphology and were encased in the ECM, which was stained with toluidine blue. The IPFP-ASCs/3D chitosan scaffolds showed positive staining for Type II collagen, proteoglycans, and Type I collagen. IPFP-ASCs showed upregulated expression of the Type II collagen, aggrecan, and SOX9 genes.	1. Chondrogenesis of IPFP-ASCs was successfully induced using a combination of TGF-β3 and BMP6 2. 3D-Printed chitosan scaffolds were delivery systems for reparative stem cells and growth factors to sites of damaged cartilage.	^183^

Biodegradable scaffolds with immobilized TGF-β3 are effective for constructing tissue-engineered cartilage. Suitable doses^170^ and scaffold carriers^171^ of TGF-β3 are important factors influencing the efficacy of the chondrogenic stimulation. Previous in vitro evidence has suggested that TGF-β3 at concentrations of 10, 20, and 60 ng·mL^−1^ exerted a dose- and time-dependent effect on the gene expression of chondrogenic markers in human MSCs.^111^ However, high doses of TGF-β (>900 ng·mL^−1^) may lead to undesired side effects such as synovitis, pannus formation, and cartilage erosion.^172^ Another in vivo study on a large animal model verified the effect of TGF-β3 and provided a reference for its suitable dose. Two hundred milliliters of blood from the jugular vein of sheep in a 25 mL suspension containing chitosan (45 mg), TGF-β3 (50 ng), and autologous ovine MSCs isolated from bone marrow to form a fibrin clot was delivered. Then, the clot was used to fill partial-thickness defects in the inner region of the patella. After 9 weeks, histological tests indicated the presence of chondrocyte‑like cells surrounded by a hyaline-like cartilaginous matrix that had wholly integrated into native cartilage.^173^ Notably, the repair results were greatly influenced by the mode of delivery. A variety of scaffolds have been explored and refined with the aim of providing compatible and optimal conditions to better mimic the natural microenvironment, which is beneficial to direct chondrogenesis. These biomaterials include poly-(lactic-co-glycolic acid) (PLGA), various hydrogels, atelocollagen, alginate beads, poly-glycolic acid (PGA), and poly-L-lactic acid (PLA).^63^ Synthetic polymers, such as PLGA, PGA and PLA, have distinct advantages in terms of shape manipulation, surface morphology, and biomechanical and biochemical properties.^174^ However, most of these polymers induce inflammatory responses in vivo.^175^ Therefore, researchers have adopted natural polymers to modify the structure of the previously used synthetic polymers. For example, Fan et al. fabricated a TGF-β3-immobilized PLGA-gelatin/chondroitin sulfate/hyaluronic acid (PLGA-GCH) hybrid scaffold and confirmed its great potential for use in cartilage tissue engineering. After implantation of an MSC-seeded PLGA-GCH scaffold crosslinked with TGF-β3, impaired cartilage and subchondral bone were successfully regenerated. Histological observation showed that chondrocyte-like cells were uniformly located within the regenerated matrix, which showed extensive metachromatic staining. Advantages of the scaffold included reduced preimplantation culture time and decreased TGF-β3 consumption.^176^ Recently, emerging evidence has focused considerable interest on the use of natural silk-derived materials owing to their unique structure, biomechanical and biochemical properties, and biocompatible features.^177^ Human MSCs were encapsulated in a 3D aqueous-derived silk scaffold and cultured in chondrogenic medium supplemented with TGF-β3 and dexamethasone. After 3 weeks, the cell arrangement and Type II collagen distribution in the constructs were similar to those of native cartilage tissues.^178^ Another similar in vivo study supported this finding. Haider et al. used genetically engineered SELP-47K in an injectable scaffold for the delivery of human MSCs cultured with or without TGF-β3.^121^ The constructs exhibited a significant increase in the content of sulfated glycosaminoglycan and total collagen, which were increased by up to 65% and 300%, respectively, with the addition of TGF-β3. With the development of material scaffolds via tissue engineering, new scaffolds based on magnetic nanoparticles crosslinked with methacrylated gelatin (GelMA), combined with slow-release TGF-β3, may be a new method to repair cartilage.^179^

Other growth factors administered with TGF-β3 can lead to synergistic chondrogenesis. For example, scaffolds loaded with TGF-β3 and bone morphogenetic protein 7 (BMP7) provided a better environment for cartilage regeneration than scaffolds loaded with either TGF-β3 or BMP7 alone.^180^ The recruitment of chondrocytes cultured with both mechano growth factor (MGF) and TGF-β3 increased significantly, by 1.8- and 2-fold in vivo and in vitro, respectively, compared with that of cells cultured with TGF-β3 alone.^181^ TGF-β3 and BMP6 are both potent chondrogenic growth factors, and their ability to induce chondrogenesis was enhanced when combined. Several studies found synergy in the chondrogenic differentiation of ADSCs, bone marrow-derived mesenchymal stem cells (BMSCs), and periodontal ligament stem cells.^182–187^ In addition, Yoo et al. put forward an innovative idea, suggesting that a combination of TGF‑β3 and BMP6, which shows a synergistic effect on chondrogenesis, loaded into an efficient carrier such as exosomes, which themselves show a chondroprotective function, offers an effective approach to treat damaged cartilage in OA.^13^ In addition to growth factors, agents such as heparin and HA enhance chondrogenesis when used in combination with TGF-β3.^188^ The synergy between HA and TGF-β3 was reported in a study in which thermoreversible hydrogels containing TGF-β3, HA and chondrocytes were implanted into mice. The mice treated with both TGF-β3 and HA exhibited a twofold increase in the production of glycosaminoglycan and collagen compared with mice treated with hydrogels alone.^189^ External factors such as the mechanical load capacity and stiffness of the substrate contribute to the regulation of TGF-β3.^190–192^

## Conclusion and perspective

Three TGF-β isoforms regulate many aspects of growth and development in a cell-type-specific manner. Although they play distinct biological roles, TGF-β3 isoforms have received particular attention because of their ability to regulate cartilage physiology and pathology. TGF-β3 is a pleiotropic cytokine involved in the whole life cycle of chondrocytes, including their proliferation, migration, differentiation, and death. Even though TGF-β3 exerts mixed inductive and inhibitory effects, TGF-β3 is considered to be show greater pro-chondrogenic effects than TGF-β1. Regulatory TGF-β3-mediated Smad2/3 and Smad1/5/8 signaling plays roles in the pathogenesis of OA. Stem cell-based tissue engineering is a relatively novel strategy to repair OA-induced cartilage lesions. TGF‑β3 is a promising therapeutic target for OA owing to its potential to regulate chondrogenesis and restore damaged cartilage. Considering recent results of various in vitro and in vivo experiments, we conclude that an optimal scaffold carrier containing stem cells and a suitable dose of TGF-β3 may form hyaline-like cartilaginous constructs that are entirely integrated into native cartilage, providing a novel approach to slowing the progression of OA.

Although considerable progress has been made in the exploration into the role of TGF-β3 in cartilage pathophysiology, some problems have yet to be solved. First, combinations of multiple growth factors/agents and scaffolds needs to be optimized. In addition, although a preclinical model has been identified, future investigations based on animal models are needed to refine this strategy, which seems a reasonable approach, and must be performed before clinical application. Furthermore, the possible complications and side effects of TGF-β3 supplementation should not be ignored. For example, high doses of TGF-β3 may lead to undesired problems such as fibrosis, synovitis, pannus formation, cartilage erosion, and osteophyte formation in cartilaginous and noncartilaginous tissues. In addition to faster and more prolific chondrogenic differentiation, supplementation with TGF-β3 is also associated with a higher susceptibility to chondrocyte hypertrophy, which may ultimately result in chondrocyte apoptosis, vascular invasion, and ossification of new tissue. Therefore, strategies to prevent hypertrophic differentiation and generate stable hyaline cartilage are absolutely necessary to cartilage tissue engineering.
